# Latent profile analysis of gambling

**DOI:** 10.3389/fpsyg.2023.1293933

**Published:** 2023-10-26

**Authors:** Şenel Çıtak

**Affiliations:** Psychological Counseling and Guidance, Department of Educational Sciences, Ordu University, Ordu, Türkiye

**Keywords:** gambling, gambling motives, latent profiles analyze, personal virtues, resilience

## Abstract

Early age of gambling onset, ease of gambling with technological developments and lack of controlling online gambling games have led to unmanageable risk of gambling. Individual-centered approaches play a significant role in managing the risk that gambling poses on public health and discerning the heterogeneity of gambling addiction. Therefore, this study employed Latent Profile Analysis (LPA), one of the individual-centered approaches, to model the interactions across the psychosocial characteristics of gamblers. The study aims to reveal the latent profiles of gambling addiction. Unlike variable-centered approaches, LPA is a contemporary technique that provides objective information regarding individual psychological processes and behaviors. The profile indicators of the study involve psychosocial characteristics such as resilience, motives to gamble (excitement/fun, avoidance, making money, socializing), purposefulness, responsibility and worthiness. Data were collected from 317 volunteers (M = 68.9%; *F* = 31.1%; mean age = 25.16 ± 6.46) through the Brief Resilience Scale (BRS), Gambling Motives Scale (GMS) and Personal Virtues Scale (PVS). The emerging profiles were defined as adventurous players (14.2%), social gamblers (9.8%), professional gamblers (32.8%), problem gamblers (24.6%) and avoidant gamblers (18.6%). The individual-centered modeling is congruent with the literature on gambling and provides a complementary perspective to understand the heterogeneous structure of gambling. The results are expected to assist mental health professionals in developing educational and clinical intervention programs for gambling behavior. Finally yet importantly, it is recommended that new LPA models be offered through the use of different indicators related to gambling addiction.

## Introduction

Considered as a behavioral addiction, gambling has become more prevalent with technological developments. Besides, individuals’ tendency toward gambling has increased during crisis periods. People mostly engage in online gambling during and after the pandemic (Emond et al., 2021). Numerous public authorities [WHO, 2017; Gambling Commission, 2022; General Directorate of Security (GDS), 2022; Green Crescent, 2022] and academic studies (Grant and Potenza, 2006; Çakıcı, 2019) reveal that gambling is a common behavior. Technological advances (accessibility) that facilitate gambling and the decrease in the age of meeting with gambling (Welte et al., 2008; Erdoğdu, 2017) demonstrate that gambling behavior has become a social risk. Online gambling games (e.g., scratchcards, lottery, bet on sports) where the age requirement is not controlled (Gambling Commission, 2022) reveal that this risk has reached unmanageable dimensions for the younger generation. It is evident that the increasing gambling behavior, the difficulty of monitoring online gambling and easy access to gambling games (scratch cards, etc.) on the streets by those under the age of 18 (Erdoğdu, 2017) will obstruct the controllability of gambling behavior and interventions. Besides, the strong relationship between gambling and alcohol addiction (Emond et al., 2021) proves that people who gamble may experience different problems (substance addiction, depression). Gambling behavior includes some psychological motives (e.g., avoidance, socialization, excitement; Lee et al., 2007; Clark, 2014). Different psychological motives result in differentiation across the individuals’ gambling profiles. Understanding the differences between the profiles of individuals who gamble may contribute to the fight against gambling (Faregh and Leth-Steensen, 2011). In this regard, it is vital to investigate the triggers of gambling habits, the reasons for starting gambling and the characteristics (profiles) of individuals who gamble in terms of reducing gambling addiction.

The characteristics of individuals who gamble (e.g., personality) are mostly associated with the etiology of gambling (Strømme et al., 2021). However, there is a dominant view that risk factors (e.g., negative characteristics such as avoidance, compensatory, socialization, making money, amusement, negative social environment) and protective factors (e.g., positive characteristics such as resilience, personal values and durability) determine the direction of problematic behaviors like gambling (Dowling et al., 2009; Hing and Gainsbury, 2013; Jang et al., 2019). The risk factors-based view claim that gambling is characterized by sensation seeking, crime, liveliness, anti-social behaviors, psychological distress (Steel and Blaszczynski, 1996) and impulsive behaviors (Lawrence et al., 2013; Dowd et al., 2020). For instance, impulsive behaviors often co-occur with addictive behaviors such as internet gaming disorder. Therefore, it is highly probable that gambling behavior has similar psychological mechanisms as internet gaming disorder and digital gaming behaviors (Iacolino et al., 2019). In addition, the etiology of gambling behavior involves a biopsychosocial structure similar to other types of addiction (Yau and Potenza, 2015). These are genetic predispositions, neurotransmitters (activation of the brain’s reward center), socio-economic factors (living in a gambling environment, desire to make easy money, entertainment, opportunities to access gambling, lack of legal restrictions) and psychological mechanisms (e.g., seeking excitement, avoiding distress, desire to socialize, cognitive assumptions such as making money in a short time, definitely winning; Lee et al., 2007; Clark, 2014; Çakıcı, 2019). In this vein, it is most probable that the symptom and problem-oriented (psychopathological) perspective of traditional psychology is dominant in seeking gambling behavior. Although studies carried out from a problem-oriented perspective eliminate the problems in a sense, the treatment and explanation levels for some disorders are considered insufficient (Jiménez-Murcia et al., 2007; Dowling et al., 2009; Merkouris et al., 2016). These evaluations have led researchers to seek answers to the question of what else can be done in cases where multidimensional psychological mechanisms such as gambling play a role. In other words, researchers have focused on positive psychology, which aims to develop the positive aspects of these individuals rather than eliminate the problems (Seligman, 2002). Therefore, the researchers should focus on identifying and developing the strong characteristics of the gambler instead of only the variables and risk factors (neuroticism, anxiety, stress, negative environment) that lead the individual to gamble. Thus, it is of great significance to determine the profiles of individuals who gamble through evaluating gambling behavior within the context of positive characteristics (e.g., resilience, values, hope, etc.) rather than psychopathological concepts (e.g., hopelessness, risk factor, anxiety, anti-sociality, neuroticism). This study is an attempt to reveal the reasons for individuals to gamble through concepts such as resilience, purposefulness, responsibility and worthiness, which are the basic concepts of positive psychology.

The causes of gambling addiction spread to a wide range within the framework of social environment, emotions, thoughts and behaviors (Buran, 2021). Cognitions (reducing stress, avoidance, desire to socialize), emotions (excitement) and behavioral variables (making money, having fun) play a critical role in individuals’ gambling behavior (Lee et al., 2007). Furthermore, individuals’ resilience skills are regarded as a protective factor on gambling behavior (Goldstein et al., 2013). Given that low self-efficacy beliefs make people more vulnerable to risky situations (gambling; Grall-Bronnec et al., 2016), it is fundamental to investigate gamblers’ resilience levels. In addition, researchers focus on positive psychology assumptions regarding the empowerment of people and their ability to adapt to changing life conditions due to the multidimensional nature of the causes that initiate and maintain gambling behavior (Lee et al., 2007; Clark, 2014) as well as the lack of the problem-focused perspective (Jiménez-Murcia et al., 2007; Dowling et al., 2009; Merkouris et al., 2016). Because resilience (Sapienza and Masten, 2011) is expected to reflect growing focus on reducing the risk factors that individuals are exposed to, adapting to changing living conditions and strengthening them. In this regard, examining the profiles of gamblers with regard to their resilience skills is predicted to shed light onto preventing gambling addiction and reducing its effects.

Current literature demonstrates that resilience skills are characterized by seeing oneself as competent, being hopeful, feeling valuable, and making decisions (e.g., taking responsibility, setting goals, making analysis; Li et al., 2018; Pinar et al., 2018). Likewise, happiness, feeling valuable, being hopeful and problem solving (being purposeful, responsible, making choices) skills aim at empowering the individual (Savi-Çakar, 2018). Purposefulness is related to the individual’s ability to make a choice in order to be happy. Values determine how an individual will behave when encountering an event/situation (Tatlıoğlu, 2014). To exemplify, individuals may act according to the instant pleasure they feel, or they may prefer the peace/pleasure as a result of their long-term behaviors (Seligman, 2002). Similarly, responsibility requires being purposeful and determined (Tatlıoğlu, 2014). Besides, feeling valuable not only contributes to mental health, but also strengthens against risky situations (Saygın, 2008). Namely, purposefulness, responsibility and worthiness have a strong effect on people’s happiness (Aydın, 2018). Hence, it may be wise to mention that positive skills such as being happy, feeling valuable, feeling responsible, being hopeful and making decisions strengthen individuals against risky behaviors like gambling addiction (Maddi et al., 2013). People seek happiness in gambling instead of in their normal lives (Lester, 1994; Wood and Griffiths, 2007), which supports these views. Therefore, investigating the positive characteristics such as responsibility, purposefulness and worthiness in terms of the profiles of individuals who gamble may provide reliable information in identifying gamblers.

Studies aiming to determine the characteristics of individuals who gamble mostly seem to have a problem-oriented perspective (Grant et al., 2014; WHO, 2017; Buran, 2021; Emond et al., 2021; Brazeau and Hodgins, 2022). These studies examined the relationships and etiological structure between gambling and excitement (Kjome et al., 2010), avoidance (Vaughan and Flack, 2021), making money, having fun (Flack and Morris, 2015), anti-sociality and risk-taking behaviors (Mishra et al., 2017). Mammadov et al. (2016) concluded that these studies are insufficient to explain the multidimensional structure of gambling behavior since they generally reflect gambling addiction in terms of problem-based variables. Since a variable-centered approach does not allow to discover the relationships across any variable and the levels of other variables, this method provides no information about individual psychological processes and behaviors (Mammadov et al., 2016). In addition, it is burdensome to distinguish precisely and effectively the heterogeneity of the target group of gamblers. Thus, studies on gambling are insufficient to reveal the problem in the literal sense (Ribeiro et al., 2021). There is a need for studies investigating gambling behavior in terms of positive psychology approach (protective factors). The relevant literature involves only two studies that explain gambling addiction within the framework of protective factors such as resilience, flexibility and positive social environment (Lussier et al., 2007; Goldstein et al., 2013). One of these studies determined subtypes of individuals who gamble through latent class analysis (CFA; Faregh and Derevensky, 2011; Goldstein et al., 2013). Hence, there is a dearth of studies identifying gambling subtypes. Moreover, more attention should be paid to the participants’ demographic differences across studies on gambling behavior. Mental disorders defined in DSM-5 are classified into types based only on the number of diagnostic criteria. The limitations of the categorical classifications adopted in the DSM-5 have been questioned by various empirical studies (Widiger et al., 2009). A mental disorder cannot be separated from others with absolute limits (Widiger and Samuel, 2005). Therefore, model-based (e.g., LPA) approaches are required to predict and distinguish the latent heterogeneity of the working group (Wang et al., 2017).

Latent Profile Analysis (LPA) is a contemporary person-centered technique used to model the characteristics of an individual (Ferguson and Hull, 2018). This method warrants that individuals in the sample belong to a unique profile–without any distinction across possible latent subgroups. Thus, the method reveals individual differences that emerge through profile membership with an individual-centered approach (Eshghi et al., 2011). Considered as a powerful technique, the latent profile suggested in the LPA enables the results to be more objective and precise by means of a set of fit indices to predict the goodness of the model (Wang and Wang, 2020). The research is unique in this respect.

Studies on gambling behavior heavily focused on factors that increase the likelihood of gambling or contribute to gambling problems; however, few studies were conducted on the probability of gambling. Therefore, there is a need for studies that examine gambling behavior in terms of positive psychology within the scope of strong statistical analyses. On the other, there is no such a study specifically published on examining individuals’ reasons for gambling and their positive characteristics (virtues such as resilience, responsibility, purposefulness, and worthiness). In this respect, this study is expected to decipher a different perspective on the subject. Herewith, it is critical to understand the profiles of individuals with gambling problems with the aim of identifying those who are most in need of gambling addiction prevention, defining the needs of individuals most at risk, and adapting early intervention strategies. This study aims at investigating the latent profiles of individuals who gamble in terms of resilience levels, reasons for gambling and positive characteristics (responsibility, purposefulness and worthiness) through the latent profile analysis method (LPA). This study aims to explore potential hidden profiles (distinct subgroups) within gambling behavior. The examination of diverse gambling profiles will enhance a better understanding of the relevant variables and existing inconsistencies in addictive behaviors such as gambling. Likewise, this study may shed light onto understanding the heterogeneity of gambling addicts and related psychological mechanisms (e.g., problem/developmental). The research hypotheses (questions) addressed in this study are as follows:

1. Do the variables affecting gambling exhibit interrelationships?
2. What are the hidden profiles within gambling behavior?

## Methods

### Participants and procedure

The study involved 415 participants selected using the criterion sampling method, which required participants to be over 18 years old and actively engaged in at least one gambling game (Ary et al., 2010). Forms completed by individuals with incomplete or incorrect information, those under the age of 18, and those not currently involved in gambling were excluded from the study. As a result, the research was conducted with a total of 317 participants. Since the data were collected from those who actively gamble, the data collection process was quite long (June 2020–December 2022). After the approval of the ethics committee (Decision dated 09 March 2020 and numbered E-2000077272), data were collected (*n* = 252) through the Google Forms link address created for potential participants and the interaction forums of online gambling and betting sites via social media (WhatsApp, Instagram, Facebook).

Besides, some of the data were collected (*n* = 65) through face-to-face interviews ith gamblers on a voluntary basis (with their consent). The ages of the participants varied across 57 to 18, and the majority of them were males (M = 68.9%; *F* = 31.1%; mean age = 25.16 ± 6.46). 39.4% of the participants were secondary education graduates, 37.1% university graduates and 25.5% primary education graduates, respectively. The participants pointed out that they gamble on platforms such as national lottery games (e.g., national lottery, sports betting), card and stone games (e.g., online poker, okey, etc.), machine games (including online), and crypto exchange (leveraged transactions). This variable was omitted from the study due to participants’ reluctance to disclose information about their professions during the pilot interviews.

### Data collection tools

#### Socio-demographic information form

Socio-demographic information form includes questions regarding age, gender, educational level and types of gambling games. This form was prepared by the researcher within the framework of the literature review on gambling addiction (Grant et al., 2014; WHO, 2017; Emond et al., 2021; Green Crescent, 2022).

#### The brief resilience scale

The tool was developed by Smith et al. (2008) to determine the participants’ resilience levels. The scale was adapted into Turkish by Doğan (2015). Being a 5-point Likert-type, the scale has a unitary construct with a total of 6 items, 3 positive and 3 negative. The results of the factor analysis performed within the scope of the validity in the Turkish adaptation study showed that the scale has a single factor structure that explains 54% of the total variance. Besides, the factor loads of the scale items were identified to differ between 0.63 and 0.79. The Cronbach alpha coefficient was found to be 0.83 (Doğan, 2015).

The Cronbach’s alpha coefficient of brief resilience scale (BRS) was determined as 0.80 in the present study. Although the Turkish version of the scale has a unitary construct, the current study determined two factors. This result is congruent with the finding that inverse items created two factors in the sample group in which low socio-economic participants were included in the study on resilience skills (Hidalgo-Rasmussen and González-Betanzos, 2019). Considering the CFA results for BRS in terms of fit indices, the factor structure for this study sample was confirmed (χ^2^/df = 0.345, RMSEA = 0.006, TLI = 0.99, CFI = 0.99; Tabachnick and Fidell, 2013). The factor loads of the items related to the scale varied between 0.56 and 0.80.

#### Gambling motives scale

The 35-item scale, designed to assess motivation underlying gambling behavior, was originally developed by Lee et al. (2007). Arcan and Karanci (2014) adapted this scale to Turkish culture. During the adaptation process, Arcan and Karanci (2014) proposed a four-subdimensional scale model for Gambling motives scale (GMS; the Turkish adaptation). Each statement were rated on a 3-point Likert type as “I agree,” “I partially disagree” and “I disagree.” The score obtained from the whole scale determines the participants’ total motivation scores regarding gambling.

The internal consistency coefficients of the factors were α = 0.83 for socialization, α = 0.78 for fun/excitement, α = 0.90 for avoidance, and α = 0.87 for monetary. The internal consistency coefficient was α = 0.92 for the whole scale. This study determined the internal consistency coefficient as α = 0.94 for the overall scale. As regards the internal consistency coefficients of the factors, α = 0.90 for socialization, α = 0.93 for fun/excitement, α = 0.91 for avoidance, and α = 0.87 for monetary. The results of CFA suggested that the scale structure was confirmed (χ^2^/df = 2.94, RMSEA = 0.01, TLI = 0.89, CFI = 0.89; Tabachnick and Fidell, 2013).

#### Personal virtues scale

The scale developed by Demirci and Ekşi (2018) consists of three factors-responsibility, purposefulness and worthiness and a total of 15 items. Each factor is scored in itself, and as the scores increase, the relevant feature also increases. The Cronbach Alpha coefficient of the whole scale was 0.86. As for the factors, the coefficients were 0.82 for responsibility, 0.77 for purposefulness and 0.75 for worthiness. The internal consistency coefficients of the scale were found to be 0.91 for the whole scale, 0.84 for the responsibility, 0.86 for the purposefulness and 0.84 for the worthiness in the present study. In addition, the fit indexes of CFA results (χ^2^/df = 3.15, RMSEA = 0.00, TLI = 0.91, CFI = 0.93) confirm the scale structure for this study (Tabachnick and Fidell, 2013).

### Data analysis

Data analysis started through examining missing data and extreme values. Of the 415 sets, missing data and the forms filled by participants under the age of 18 and those who do not currently gamble were excluded from the dataset, and analyses were conducted on the dataset, which included 317 participants. Data were examined for normality of distribution, multicollinearity, multivariate normality and linearity. In this regard, all assumptions were met. Afterwards, confirmatory factor analysis was performed to reveal the structure of the measurement tools. Latent profile analysis (LPA) was conducted via the R program related to the variables of resilience, reasons for gambling, responsibility, purposefulness and worthiness.

Latent profile analysis is a statistical procedure in which latent indicators are used continuously while performing latent class analysis (Rosenberg et al., 2018). LPA analyses were made by determining which models (EEI, EEE, VVI, VVV…) defined the best profile memberships. In this context, the analyses made via the R programming language (Mclust) demonstrated that the VVV (varying volume, varying shape, varying orientation) model defines the best profile memberships (Wardenaar, 2021). Hence, five profile memberships were identified within the framework of the criteria (BIC, AIC, BLRT, Entropy) obtained from the analyses made through the use of TidyLPA and Mclust packages (Rosenberg et al., 2018).

Thereafter, the number of latent profiles within the working group was determined in LPA. BIC, AIC, LMR-LRT, entropy value and posterior probabilities were used to determine the participants’ latent profile number. Smaller values for AIC and BIC indicate a better fitting model. Entropy is an index for predicting the quality of class assignments, and a higher entropy refers to a higher classification precision (Rosenberg et al., 2018). Besides, a nonsignificant (*p* > 0.05) LMR-LRT value signifies that adding more profiles to the model does not improve the model. Moreover, a value close to 1.0 for entropy values points a better decision on the number of profiles to include (Wang and Wang, 2020). Finally, the differences across the variables and latent profiles were examined through ANOVA and Chi-square analyses.

## Findings

Normality tests were initially performed on the data (Skewness and Kurtosis), and hence the data showed a normal distribution (+1; −1; Tabachnick and Fidell, 2013). The relationship across the variables was examined ([r ≥ 0, low, r ≥ 0.3 medium, and r ≥ 0.5] Cohen, 1994). Table 1 displays the correlation summary for the research variable.

**Table 1:** Correlations, means (m), and standard deviations (sd) related to variables.

Variable	1	2	3	4	5	6	7	8	M	SD
Resilience	-								19.41	4.75
Excitement/fun	0.76	-							45.67	12.70
Avoidance	−0.35^**^	0.29^**^	-						17.80	7.23
Making money	−0.01	0.45^**^	0.48^**^	-					22.10	7.91
Socialization	0.02	0.46^**^	0.40^**^	0.41^**^	-				16.51	6.60
Responsibility	0.32^**^	0.96	−0.22^**^	0.16^**^	0.05	-			19.58	3.84
Purposefulness	0.33^**^	0.19^**^	0.17^**^	0.16	0.13^*^	0.54^**^	-		18.66	4.34
Worthiness	0.35^**^	0.16^**^	−0.20^**^	0.05	0.11^*^	53^**^	0.65^**^	-	18.16	4.20

^*^p < 0.05; ^**^p < 0.01.

The presence of a relationship between resilience and personal virtues (responsibility, purposefulness and worthiness) is an unexpected situation since the sample consists of those who still report gambling, and GMS does not determine the level of gambling, but aims to identify the types of gambling motives of current gamblers. For instance, it is experiential and theoretically unlikely that an increase in resilience will increase one’s motivation to gamble for socialization. The relevant literature suggests that risky behavior is expected to decrease as resilience increases (Sapienza and Masten, 2011). Therefore, this study did not analyze the relationship across the variables representing the positive characteristics of individuals and the reasons for gambling as the severity of gambling was not measured. However, all variables were analyzed together and displayed below. Accordingly, no relationship was identified between resilience and the factors of excitement/entertainment, making money and socializing, while a negative relationship was found across avoidance factor. A positive correlation was determined between resilience and responsibility, purposefulness and worthiness (*p* ≤ 0.001). While a relationship was noted between responsibility, avoidance and making money, excitement/entertainment and socialization were free from any significant relationship. A relationship was found between purposefulness and worthiness and excitement/fun, avoidance (*p* ≤ 0.001) and socialization (*p* ≤ 0.05); whereas no relationship was determined across making money. Besides, a positive relationship was found between excitement/entertainment, avoidance, making money and socializing (*p* ≤ 0.001).

LPA was performed after the correlation analysis for the main variables for providing a much more detailed description of the profiles of gamblers (Wang et al., 2017). Findings regarding the analyzes and model fit of the LPA analysis are depicted in Tables 2, 3 (EEI) and (EEE).

**Table 2:** EEI model fit statistics for determining the optimal number of classes.

Model	LL	BIC	AIC	BLRT	BLRT (*p*)	Profile comparisons	Entropy
1	−3,651	7,334	7,395	340.18361	0.001	1 vs. 2	1
2	−3,481	7,012	7,107	171.10886	0.001	2 vs. 3	0.787
3	−3,396	6,859	6,987	147.10182	0.001	3 vs. 4	0.817
4	−3,322	6,730	6,892	64.46335	0.001	4 vs. 5	0.876
5	−3,290	6,684	6,880	106.14946	0.001	5 vs. 6	0.866
6	−3,237	6,595	6,826	53.70494	0.001	6 vs. 7	0.867

**Table 3:** EEE model fit statistics for determining the optimal number of classes.

Model	LL	BIC	AIC	BLRT	BLRT (*p*)	Profile comparisons	Entropy
1	−3,240	6,568	6,734			1 vs. 2	1
2	−3,229	6,565	6,765	20.61364	0.199	2 vs. 3	0.676

All psychosocial variables (resilience, responsibility, purposefulness, worthiness) and reasons for gambling (excitement/fun, avoidance, making money, socializing) were included in the analysis. Analyses were conducted via TidyLPA and Mclust packages over the R program (Rosenberg et al., 2018). LPA determined which models identified the best profile memberships. Akaike information criterion (AIC), Bayes information criterion (BIC), adjusted Bayes information criterion (aBIC), entropy and bootstrap likelihood ratio test (BLRT) were accepted as the basic criteria of model fit (Wang and Wang, 2020). EEI, EEE, VVI, VVV models (Fraley et al., 2012) defining good profile memberships were tested for gradual inclusion of latent profile memberships. All models (except for EEE) except for the VVV (varying volume, varying shape, varying orientation) model have consistently offered to add a new profile membership to the model. The EEE (equal volume, equal shape, and equal orientation) model offered only three profile memberships. The VVV model, on the other, suggested the five-profile structure and the LMR-LRT values (*p* > 0.05) showed that adding more than five profiles to the model did not improve the model fit. Other models do not meet these criteria. In addition, entropy values (closer to 1.0), AIC and BIC values (preferring a smaller value) confirm the fit of this model (Masyn, 2013; Wang and Wang, 2020).

The number of profiles was determined according to BIC, AIC, BLRT and entropy value in the final model. Information on model fit information is summarized in Table 4.

**Table 4:** Final model (VVV) fit statistics for determining the optimal number of classes.

Model	LL	BIC	AIC	BLRT	BLRT (*p*)	Profile comparisons	Entropy
1	−3,240	6,734	6,568	323.5066	0.001	1 vs. 2	1
2	−3,078	6,970	6,334	148.3214	0.001	2 vs. 3	0.958
3	−3,004	6,782	6,276	142.6273	0.004	3 vs. 4	0.907
4	−2,933	6,899	6,223	261.5832	0.001	4 vs. 5	0.872
5	−2,802	6,897	6,052	108.0877	0.068	5 vs. 6	0.923

The AIC value is the smallest for the five-profile solution (6052), while the BIC is the smallest for the two-profile solution (6734). The smallest BIC value after the two-profile model was observed within the five-profile model. Likewise, the highest entropy value (0.95) refers to the three-profile model. However, BLRT values [BLRT(*p*)=0.068] indicate that adding a profile to the model with five profiles will increase the model fit and adding a profile to the model when the sixth profile membership is added does not increase the fit (Ramaswamy et al., 1993). Thus, the five-profile was chosen as the most appropriate model based on all indices.

**Figure 1:**
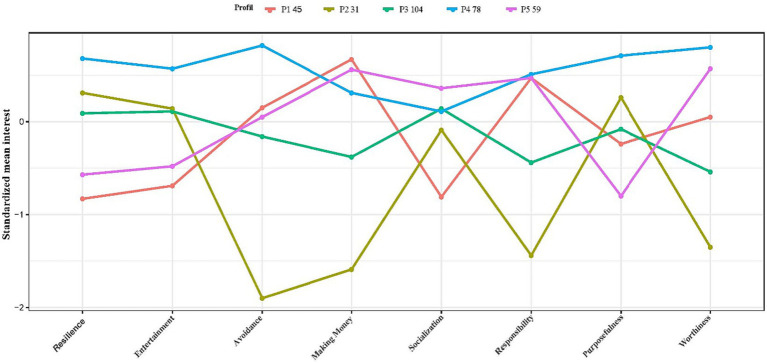
The latent profile model of gamblers.

The profiles were entitled as descriptors to increase the readability of the data in Table 4. Figure 1 presents these profile memberships.

Based on these definitions, Profile 1 defines people with the highest level of resilience and purposefulness, and medium level of responsibility and worthiness. These people also gamble for entertainment, socialization, and winning money at a very high level (compared to other profiles), yet they have low levels of avoidance (together with profile 2). This profile (*n* = 45, 14.2%) was defined as adventurous players. Profile definitions were established by drawing upon relevant literature (Ciarrocchi, 2002; Faregh and Leth-Steensen, 2011; McCormack and Griffiths, 2012; Weinstock et al., 2013; Çakıcı, 2019) and incorporating the researcher’s observations.

Profile 2 is similar to profile 1 and has lower scores (*n* = 31, 9.8%). This profile includes individuals with a medium level of resilience and purposefulness scores and the lowest scores for responsibility and worthiness. They also gamble with high levels of entertainment and socialization (*n* = 31, 9.8%; low by profile 1, high by others), whereas the lowest level of avoidance and money-making gamblers. This profile was called social gamblers.

Those in Profile 3 have the lowest scores for resilience skills and purposefulness (along with profile 5) and those with high responsibility and worthiness scores (lower than Profiles 1 and 5). They also have the lowest entertainment (along with profile 3), average avoidance and socialization, the highest money-making gambling scores. This profile (*n* = 104, 32.8%) was conceptualized as professional gamblers.

Profile 4 has an average score in terms of resilience, worthiness and responsibility skills, while individuals in this profile have higher scores related to purposefulness (lower compared to profile 1). These individuals have average gambling scores due to entertainment, making money, avoidance and socializing. This profile (*n* = 78, 26.6%) was categorized as problem gamblers.

Similar to Profile 3, individuals in Profile 5 have the lowest scores for resilience and purposefulness, while the highest scores for responsibility and worthiness. They also have the lowest entertainment (along with profile 1), average money-making and socializing gambling scores, and the highest avoidance (lower than profile1) gambling scores. This profile (*n* = 59, 18.6%) was defined as avoidant gamblers.

Table 5 depicts the comparison across the profiles in terms of age, gender and educational level. Analyzes regarding profile memberships are presented as follows.

**Table 5:** Descriptive data and analysis of variance across profiles.

	M (SD)						ANOVA			
	Profile 1	Profile 2	Profile 3	Profile 4	Profile 5	Overall				
	*n* = 45	*n* = 31	*n* = 104	*n* = 78	*n* = 59	*n* = 317				
Variable	(14.2%)	(9.8%)	(32.8%)	(24.6%)	(18.6%)	(100%)	*F*(6,47)	*p*	*η* ^2^	Scheffe
Resilience	23.83	20.29	17.96	18.43	19,65	19.45	12,48	0.000	0.14	2 = 3 = 4 = 5 < 1
	(5,80)	2.92	2.22	5.47	2,91	4.68				
Excitement	53.03	37.31	42.65	45.13	52,44	45.57	15,77	0.000	0.17	2 = 3 < 4 < 5 = 1
	13.03	12.64	5.58	13.29	9,67	12.67				
Avoidance	14.40	13.17	21.52	17.94	19,80	17.68	13,92	0.000	0.15	2 = 1 < 4 < 5 = 3
	8.99	4.63	2.68	8.18	4,86	7.15				
M. money	23.11	15.17	23.73	21.84	27,13	22.11	20,41	0.000	0.21	2 < 4 = 1 = 3 < 5
	10.17	5.33	3.04	8.31	5,42	7.87				
Socialization	17.23	10.06	18.62	17.22	18,87	16.57	20,41	0.000	0.20	2 < 1 = 3 = 4 = 5
	7.94	2.99	2.68	7.08	5,23	6.51				
Purposeful…	24.66	18.65	16.52	18.79	17,07	18.75	31,47	0.000	0.29	2 = 3 = 5 < 4 < 1
	0.68	4.18	1.94	4.08	4,09	4.23				
Responsible…	24.37	20.02	17.00	20.82	15,44	19.54	79,48	0.000	0.50	3 = 5 < 2 = 4 < 1
	0.84	3.06	2.43	2.82	2,99	3.78				
Worthiness	24.63	19.02	16.31	17.62	16,35	18.18	41,88	0.000	0.35	3 = 4 = 5 < 2 < 1
	0.69	3.29	1.66	3.85	4,30	4.14				
Age	24.14	26.58	25.10	25.59	23.73	25.20	1,67	0.015	0.02	1 = 2 = 3 = 4 = 5
	5.05	7.14	5.93	7.34	4.34	6.46				
Female	2.20%	6.20%	3.70%	12.40%	6.50%	31.10%	-	-	-	-
Male	8.70%	9.90%	12.40%	27.30%	10.60%	68.90%	-	-	-	-
S. school	4.00%	3.10%	5.60%	8.40%	4.30%	25.50%	-	-	-	-
H. school	3.70%	5.90%	5.30%	16.10%	8.40%	39.40%	-	-	-	-
University	3.10%	7.10%	5.30%	15.20%	4.30%	35.10%	-	-	-	-

^*^p < 0.05.

ANOVA results revealed no significant difference across the age profiles of the individuals. Besides, chi-square tests of independence showed no significant difference between profile membership and gender (χ2(4) = 6.18, *p* < 0.018; Cramer’s V = 0.201) and educational status [χ2(8) = 11.20, *p* = 0.019; Cramer’s V = 0.13]. Upon examining Table 5 in terms of all variables (age, gender, educational level), the problem gambler profile membership (profile 4) was found to have a higher probability.

## Discussion

Based on a person-centered perspective (LPA), this study investigated different gambling profiles by including psychosocial indicators such as reasons for gambling, resilience, responsibility, purposefulness and worthiness. The results are congruent with those in the current literature (Ciarrocchi, 2002; Ögel, 2010; McCormack and Griffiths, 2012; Weinstock et al., 2013; Çakıcı, 2019). The study identified seven different gambling profiles. Although there is no such a study specifically published on profile definitions made through LPA, the relevant literature involves definitions based on LCA (eight-class structure; Faregh and Leth-Steensen, 2011). The study conducted by Raybould et al. (2022) defined four to six different classes. Likewise, some studies defined gambling addicts as adventurous, avoidant, social and problem gamblers (Ciarrocchi, 2002; Çakıcı, 2019). The literature also includes studies that associate gambling addiction with personality structures (Vaddiparti and Cottler, 2017). The inadequacy of variable-centered approaches in explaining the multidimensional structure of gambling reveals the power of the results of this study and these profiles may provide ideas regarding individual gambling behavior (Mammadov et al., 2016). Considering the results of the study, different groups of gambling may be defined and new insights may be provided for gambling behavior.

Profile 1, in which gambling behavior was first defined, refers to individuals with the highest levels of resilience and purposefulness, but with medium level of perceptions toward responsibility and worthiness. They gamble at a very high level of fun, socialization and making money and low level of gambling for avoidance. The finding that resilient people are more optimistic and have strong self-efficacy beliefs (Folkman and Lazarus, 1985) suggested that people with high resilience gamble more for entertainment and money. Because people who are self-confident and have positive expectations may feel that they can earn more money. Similarly, it is unexpected for a person with high self-efficacy to engage in avoidance behavior (Bond and Flaxman, 2006). In addition, low level of addictive behaviors (e.g., alcohol) in individuals with high resilience points that people with this profile may also play gambling games for entertainment (Green et al., 2014). In this regard, the first profile was defined as adventurous gamblers (Ciarrocchi, 2002; Ögel, 2010; Çakıcı, 2019). Overall, the educational and clinical implications underline the need for holistic diagnostics that will allow the individualization of intervention approach (Orbach and Fritz, 2022). Thus, a different intervention focus can be used for each profile. To illustrate, emotion regulation strategies can be used to reinforce feelings of responsibility and worthiness for the adventurous profile. In particular, Positive Cognitive Behavioral Therapy strategies (e.g., benefit-harm analysis) based on taking responsibility and self-evaluation can be used (Leahy, 2010). Such studies may help people determine their potential and make them feel better (Bannink and Jackson, 2011). Behavior experiments may be planned in line with alternative purposes to change the excitement/entertainment motives such as making money. As people’s conscious awareness and sense of worthiness increase, their tendency toward dysfunctional experiences (making money, gambling for excitement) may decrease (Beck, 2020).

Similar to the adventurous gamblers (profile 1), those in profile-2 were identified to have a medium level of resilience and purposefulness, but low level of responsibility and worthiness. Even though gambling addiction is risky, individuals continue to gamble. This may be explained by the ability to recover and adapt (resilience) as resilience also provides easy adaptation to mistakes despite risky experiences such as money losses (Sapienza and Masten, 2011). Likewise, the ability to be purposeful reduces their risk of inclining to pathological gambling (Derevensky, 2012). They were also determined to gamble for socialization at a high level, exhibit avoidance at the lowest level and play games for the purpose of winning money. People with low worthiness beliefs may join risky social groups for socialization and exhibit submissive behaviors to group members (Verheul, 2001). Given that individuals in this profile gamble with the motivation of being in social environments and making friends (Yip et al., 2011), they should be conceptualized as social gamblers (Ciarrocchi, 2002; Yip et al., 2011; Çakıcı, 2019). The lack of motivation for financial gain (Macit, 2021) supports the structure of the profile of social gamblers (Lee et al., 2006). When the level of resilience and purposefulness are high, the possibility of pathological gambling may decrease. Because individuals with low self-efficacy may develop motivation to gamble (avoidance, socialization, etc.) to meet multiple psychological needs (Weinstock et al., 2013). Besides, childhood abuse, neglect or trauma may trigger gambling (McCormick and Taber, 1987) since neglect and abuse can often develop worthlessness in people (Matthews, 2013). When people do not feel themselves as valuable, they compensate for their social anxiety by choosing experiences/situations that they feel approved by other people (gambling, internet gaming) through games (Verheul, 2001). Besides, they may gamble because of their friends or loneliness (Trevorrow and Moore, 1998). Not belonging to a community or being excluded from society and feeling alone as well as the desire to socialize with others may increase gambling behavior (Bullen and Onyx, 1998). Individuals who display gambling behavior were identified to have self-discipline problems related to stopping these habits, and they attempted to create a social environment through gambling in order to compensate for their decision-making problems and low self-esteem (low sense of worthiness; Derevensky, 2012, p. 70). The reflection of low responsibility skills in gamblers to life may be through cognitive distortions such as personalization. Considering that people avoid taking responsibility due to their social anxiety (Beck, 2020), the definition of social gamblers is in line with the current literature. Therefore, various studies on responsibility skills and worthiness beliefs may be carried out while preparing individual-centered prevention and intervention programs for people in the profile of social gamblers. To exemplify, assignments may be provided for collaborative work or behavior experiments may be created to increase responsibility-taking skills (Türkçapar, 2021). Besides, studying on cognitions such as worthlessness (e.g., reconstructing, generating alternative thinking, seeking evidence) may help reduce individuals’ psychopathological susceptibility (Dozois et al., 2009). Social gamblers’ self-confidence increases thanks to successful assignment since the probability of performing homework or behavior experiments will increase thanks to high endurance skills (Fennell et al., 2004). Hence, the sense of responsibility and worthiness may be strengthened.

Unlike social gamblers, those who are defined as Profile-3 had low resilience and the ability to act purposefully, yet medium level of responsibility and worthiness. They were identified to mostly gamble with the aim of making money. These results indicated that individuals in this group do not consider gambling as a means of entertainment. Moreover, people in this profile gamble for socialization and avoidance, albeit limited. Those with high motivation to gamble for money making are defined as professional gamblers in the relevant literature (Ögel, 2010; McCormack and Griffiths, 2012; Weinstock et al., 2013). People who gamble for winning money attempt to gamble in order to regain their possible money losses (Morehead, 1950). Because those with high worthiness are expected to feel valuable, to be forgiving and understanding toward themselves, and not to criticize themselves ruthlessly. They can be understanding and forgiving especially in situations such as losing money in gambling or having an argument with their social environment (mother/father, spouse) due to gambling (Demirci and Ekşi, 2018). Since these self-evaluations will make people happy, they are likely to continue gambling. Even if they achieve their goal of winning money, they make more attempts to earn more (Weinstock et al., 2013). They cannot adapt to new conditions (low resilience) in serious money losses, and may engage in impulsive behaviors (suicide, fight, leaving home, etc.; Cenan, 2008). It is conceptually consistent to define the people in Profile-3 as professional gamblers. In addition, their self-perceptions of efficacy (worthiness) may increase their belief that they gamble well and may trigger more gambling behavior. The fact that professional gamblers prefer games that require certain skills and proficiency rather than gambling games where the luck factor is at the forefront may be an indicator of the high level of self-efficacy perceptions (Hing et al., 2015). That responsibility is generally characterized by competence (worthiness; McCrae and Costa, 1991) and mostly observed in success-oriented people (Vollrath, 2001) may explain the finding in regards to a high level of responsibility in profile characteristics. Therefore, they are not expected to blame themselves when they lose money (responsibility), and this process can increase their motivation to gamble for money (Weinstock et al., 2013). This may also explain the low level of gambling behavior for entertainment and socialization. The relation between responsibility and happiness (McCrae and Costa, 2003, p. 164–179). is in conjunction with gambling for avoidance. Despite a limited extent, avoidant gambling behavior may be related to the possible stress experienced after losing money (Jacobs, 1986). Considering the relationship between the discipline, commitment and coping skills of professional gamblers (Rosecrance, 1988, p. 221) with resilience (Kasapoğlu, 2020), profile definitions are considered to be consistent with the literature. Their preference for skill-based games (Hing et al., 2015) may also be related to their sense of worthiness. Although these competencies of professional gamblers seem positive, they may increase the risk of gambling addiction (Monaghan et al., 2008). Professional gambling is promoted by celebrities and the media as a legitimate profession, and hence motivating young people to be a professional gambler (Monaghan et al., 2008). Therefore, prevention and intervention studies are of great importance for professional gamblers. It would be useful to create a framework for prevention and intervention studies by taking the profile characteristics of professional gamblers into account (Barnicot et al., 2012). In this regard, motivational interviewing techniques can be used on the basis of the high value and responsibility levels of professional gamblers (Ögel, 2009). In cases where shame and stigmatization thoughts are dominant, self-help-based intervention studies may be preferred by conducting awareness studies on the problems caused by gambling (Hodgins et al., 2001). Alternative ways of making money and benefit-harm analyses of gambling may be sought by considering such individuals’ competencies (Ögel, 2009). For individuals interested in recreational gambling, enhancing their motivation for future endeavors can be achieved by developing effective leisure activities. This approach may help counter potential resistance to treatment (Mannino and Caronia, 2017). Leisure activities may be planned against the possibility of increasing resistance to treatment in individuals who gamble for fun. As people’s boredom tendencies increase, their tendency toward also increases (Yang et al., 2020). The most significant challenge in the prevention and intervention studies of people with this profile may result from the society’s positive acceptance of the concept of professional gambler (Hing et al., 2015). Informative public health studies may be conducted on gambling problems and the pathological transformation of gambling in cases where professional gambling is welcomed positively by the society (Monaghan et al., 2008). This study revealed that profile-4 held the characteristics of problem gamblers. This habit of professional gamblers is likely to turn into a gambling addiction problem (Monaghan et al., 2008). Profile-4 consists of those who have average resilience, responsibility, purposefulness and worthiness scores. In addition, they gamble for entertainment, making money, avoiding and socializing. It may be wise to mention that these people possess many sources of motivation to gamble, which makes them likely problem (heavy) gamblers (Petry et al., 2005; Nowak and Aloe, 2014) since having many motives for gambling can make it difficult for people to control their daily life activities. Losing control of daily life activities indicates that the individual is a problem gambler (Ögel, 2010). Individuals in this profile can be defined as problem gamblers because this type of gambling is considered to weaken their psychosocial functions (Weinstock et al., 2013). Likewise, the low self-efficacy of problem gamblers (Weinstock et al., 2013) is in line with profile characteristics. Similarly, problem gamblers with low self-efficacy may exhibit increased susceptibility to risky behaviors. Moreover, individuals who engage in gambling activities are driven by various motivations, including financial gain (Weinstock et al., 2013), escapism, enjoyment (Cenan, 2008), excitement, risk-taking, and competition (Nowak and Aloe, 2014). These factors contribute to the characterization of profile-4 as a problem gambler. This alignment between profile-4’s attributes and the existing literature underscores the accuracy of the profile definition (Weinstock et al., 2013). Considering that problem gamblers have a medium level of resilience, responsibility, purposefulness and worthiness beliefs, prevention and intervention studies, which are at the center of positive psychology (e.g., Positive CBT), may be carried out along with the problem-focused approaches used in the treatment of problem gamblers. The high level of purposefulness skills, albeit limited, may contribute to the planning and completion of new daily life activities. Because individuals with high purposefulness skills set new goals for themselves and have high motivation to work for these goals (Tatlıoğlu, 2014). In addition, other prevention and intervention programs that are considered to have an impact on the development of individuals may be analyzed (Cowlishaw et al., 2012; Yakovenko et al., 2015). Given problem gamblers are the most risky group (Hing et al., 2015), individual-centered prevention and intervention studies are believed to become much more necessary. As problem gamblers tend to show themselves as professional gamblers and feel ashamed of their treatment, their motivation toward prevention and intervention options is low (Hing et al., 2015). As regards the high probability of being a member of the problem gambler profile, it suggests that the problem gambler profile is more common among individuals (Steel and Blaszczynski, 1996; Weinstock et al., 2013; Nowak and Aloe, 2014). Hence, it is evident that it poses a risk to public health. Individual-centered planning of prevention and intervention studies is required especially when the high rate of withdrawal from treatment by problem gamblers (Melville et al., 2007) and other risky behaviors accompanying these individuals (Weinstock et al., 2013) are taken into account (Barnicot et al., 2012). The results highlighted that profile-5 involves those who have low level of resilience and purposefulness and who have the highest sense of responsibility and worthiness. The present study also revealed that people in profile-5 mostly gamble for avoidance and the least for entertainment. Participants in Profile-5 reported an average level of socializing and money-making gambling. This profile encompasses avoidant gamblers whose gambling motives are to release tension and anxiety in their lives (Clarke, 2008; Çakıcı, 2019). Mental procrastination is provided by focusing on a specific issue for cognitions that cause anxiety and stress in avoidance behavior (Freeston et al., 1996). Situations such as the sounds of slot machines when they reach a high profit level, satisfying social activity hunger or spending time increase avoidance gambling (Jacobs, 1986). Poor coping skills in people who gamble for avoidance (Gupta et al., 2004) can be explained by low resilience. People with high anxiety tend to turn to a negative process as they cannot trust their problem-solving skills. Thus, they tend to avoid problems (Davey, 1994; Iacolino et al., 2019). Since the lack of resilience will result in the inadequacy of coping skills (e.g., coping with guilt), it is most probable that avoidant gambling elicits pathological gambling (Yi and Kanetkar, 2011). Those who gamble for avoidance also reported that they gamble for monetary purposes and socializing, meaning that the profile structure is defined correctly (Case and Olino, 2020). Besides, avoiding people due to unfortunate situations or believing in one’s own luck and competence (worthiness) indicates the high level of control (responsibility) in this profile (Wood and Griffiths, 2007). The high level of control feelings may provide information about the level of individuals’ responsibility and worthiness beliefs (Pekrun, 2006). Therefore, profile characteristics conceptualized as avoidant gamblers are parallel with those available in the literature (Clarke, 2008; Çakıcı, 2019; Case and Olino, 2020). Just as it is not desirable for people to gamble in order to avoid stressful situations, regret resulting from possible monetary losses and negative mood caused by avoidance as a result of repetitive gambling often transforms avoidant gambling behavior into pathological gambling (Blaszczynski and Nower, 2002; Marotta, 2002). In this vein, individual-centered prevention and intervention studies have gained significance for avoidant gamblers (Barnicot et al., 2012). In particular, avoidants’ strong feelings of control (responsibility and worthiness) may be taken into the focus of activities to be carried out in prevention and intervention services. For instance; avoidant gamblers often experience the lack of social activity in their life as a means of avoiding gambling (Wood and Griffiths, 2007; Yang et al., 2020). Therefore, it may be easier for people with strong feelings of control to plan social activities. Thus, they experience an activity different from gambling as an avoidance behavior. In this regard, there may be a different way out of gambling and individuals’ sense of purposefulness may develop. Different ways of avoiding, socializing, spending time and earning money may be suggested, and benefit-harm analyses of these activities can be conducted as the opposite strategy of suppressing thought is to raise awareness (Lavender et al., 2009). In addition, increased awareness levels are effective in reducing gambling addiction for those who gamble frequently (Lakey et al., 2007). In this context, mindfulness-based stress reduction (acceptance and commitment therapy) techniques may be preferred (Hayes et al., 2006). Similarly, unhealthy coping efforts with anxiety may lead to the development of anxiety disorder in people (Orcutt et al., 2005). Currently, the most widely practiced evidence-based treatment for gambling addicts is construed as cognitive-behavioral therapy, which focuses on reconstructing gambling-related dysfunctional cognitions (Toneatto, 2002). Therefore, Positive CBT strategies (exposure against avoidance) may be used by focusing on the positive characteristics of avoidant gamblers. Although CBT seems to be effective for some individuals, the high rates of unresponsiveness and relapse to treatment reveal the need to consider alternative treatment approaches (Toneatto et al., 2007). In this sense, different individual-centered treatments can also be applied because gamblers’ lack of motivation to seek help and their cognition that treatments will not help (Gainsbury et al., 2014) can put them in a vicious circle. Since the hopelessness created by this cycle (Matthews, 2013) can be overcome by individualization of treatment, it is vital to uncover gambling profiles (Barnicot et al., 2012). Gambling profiles created in this context are expected to make a serious contribution to the prevention and intervention studies of gambling addiction. Moreover, awareness-raising studies may be conducted with regard to the profiles of gamblers, to encourage the treatment process, stigma, shame, denial, and help-seeking (Gainsbury et al., 2014).

## Results and limitations

This study revealed five different profiles of gambling addiction. The results demonstrated that gambling profiles were conceptualized as adventurous gamblers, social gamblers, professional gamblers, pathological and avoidant gamblers. However, this study holds some limitations. First, the study was based on voluntary self-reports since the sample consisted of individuals who gamble. Therefore, multi-method research designs that will eliminate the social acceptance created by self-report may be a useful approach in this regard. Secondly, the generalizability of the study was limited as it was conducted only with the sample in Turkey and mainly consisted of male participants. Likewise, the data consisted of only the statements of the participants. Therefore, further studies with larger sample groups may be carried out in coordination with associations working on addictions. Third, measurements were made in terms of resilience, reasons for gambling, and personal virtues via LPA. LPA models including other protective and risk factors for gambling addiction may be developed for future researchers to explore various dimensions and associated variables. Several studies that reveal cause-effect relationships across variables (e.g., longitudinal) may be planned to develop and validate individualized treatments according to gambling profiles and to reduce the limitations of gambling-related profiles. The study evaluated that the profiles obtained through LPA will transform the heterogeneous structure of gambling behavior into a more understandable phenomenon (Wang et al., 2017). Because the success level of prevention and intervention studies for gambling behavior varies depending on the heterogeneity of gamblers (Merkouris et al., 2016). Therefore, the results offer a holistic assessment in understanding gambling behavior. Individual-centered prevention and intervention approaches may be developed to reduce the limitations of existing prevention and intervention programs (Echeburúa et al., 2001; Hodgins et al., 2001) through the gambling profiles thus obtained. Diagnostic explanations (Barnicot et al., 2012) can make a significant contribution to reducing gambling behavior rather than an intervention that will allow the analysis of heterogeneous phenomenology and the customization of the intervention approach toward this structure. Besides, psychoeducational studies, educational and clinical implications for the prevention of gambling addiction can be obtained with regard to gambling profiles. Psycho-educational studies will be beneficial in terms of providing individual social support, improving resilience, ensuring responsibility to gamblers, being purposeful and feeling valuable as well as raising awareness. In addition, these results may provide significant contributions to those working in schools and university psychological counseling centers (e.g., prevention), policy makers (e.g., access to gambling) and researchers (developing individual-centered approach models on gambling).

## Data availability statement

The raw data supporting the conclusions of this article will be made available by the authors, without undue reservation.

## Ethics statement

The studies involving humans were approved by Atatürk University Rectorate: (Decision dated 09.03.2020 and numbered E-2000077272). The studies were conducted in accordance with the local legislation and institutional requirements. The participants provided their written informed consent to participate in this study.

## Author contributions

ŞÇ: Conceptualization, Data curation, Formal analysis, Funding acquisition, Investigation, Methodology, Resources, Validation, Visualization, Writing – original draft, Writing – review & editing.
